# Drawing lessons from the standard treatment guidelines and essential medicines list concept in South Africa as the country moves towards national health insurance

**DOI:** 10.4102/safp.v63i1.5145

**Published:** 2021-01-12

**Authors:** Velisha A. Perumal-Pillay, Fatima Suleman

**Affiliations:** 1 Discipline of Pharmaceutical Sciences, College of Health Sciences, University of KwaZulu-Natal, Durban, South Africa

**Keywords:** essential medicines lists, health systems strengthening, national health insurance, South Africa, standard treatment guidelines

## Abstract

The essential medicines concept is recognised as an instrument to improve medicines access and to promote cost-effective use of health resources. South Africa adopted the concept and implemented the *Standard Treatment Guidelines and Essential Medicines List* (STGs/EML) in 1996 when the National Drug Policy for South Africa was launched. The STGs/EML was meant to address the inequities in medicines access and use and to ensure a standard of care to all citizens, yet these inequities still exist. The implementation of the new National Health Insurance (NHI) scheme is envisaged to relieve this healthcare inequity. The STGs/EML still forms the basis of care in the public sector, but a critique of implementing this tool and lessons that can be applied from this implementation for NHI are lacking. This piece addresses these shortfalls and highlights questions surrounding the implementation of the STGs/EML.

## Introduction

The essential medicines concept is internationally recognised as an instrument to improve medicines access and use and to promote the cost-effective use of health resources. It guides countries with limited resources to better manage healthcare services and provides a means to maximise limited available resources.^1^ The careful selection of a limited number of essential medicines leads to better medicine management and improved quality of care. The essential medicines list is a fundamental tool that guides countries in the procurement and distribution processes, which ultimately reduces costs to both the healthcare system and the patient. Representative essential medicines policies are key to supporting health and attaining sustainable development.^1,2^

## The essential medicines concept and South Africa

South Africa adopted the essential medicines concept in 1996, when the country’s new democratic government launched the National Drug Policy for South Africa. One of the objectives of this policy was for the ministerially appointed Drug Policy Committee to create an essential medicines list for use in the public sector and to prepare treatment guidelines for healthcare personnel. By 1994, a total of about 2600 medicines were being purchased in the public sector.^3^ The list contained a large number of examples from the same pharmacological classes, which reflected the personal preferences of those responsible for selection, rather than a deliberate choice between interchangeable options. The creation of the first National Essential Drugs List Committee (later renamed the ‘National Essential Medicines List Committee’, or NEMLC) actually preceded the publication of the national medicines policy.^4^ One of the first tasks of the NEMLC was to rationalise the list of medicines in the public sector and guide procurememt according to evidence-based treatment guidelines.

The resultant *Standard Treatment Guidelines and Essential Medicines List* (STGs/EML) was first published in 1996 at the primary healthcare level, then secondary and tertiary hospital levels in subsequent years, with separate editions for adults and paediatrics. The STGs/EML was meant to address the health objectives of the National Drug Policy, which essentially were intended to ensure the availability and accessibility of safe, efficacious and quality essential medicines to all citizens. By 1998, a far more restricted list of 337 medicines (in 422 dosage forms) had been compiled, which compared well with the *WHO Model List of Essential Medicines.*^4^ It was envisaged that the implementation of the STGs/EML would address the inequities in access to healthcare and medicines inherited from the apartheid government by ensuring a standard level of care to all citizens seeking care in a public sector healthcare facility,^5^ but this has not happened and healthcare is still inequitable. However, the concept of the EML is still relevant and has been incorporated into universal health coverage (UHC) principles as well.

## Access to medicines and universal health coverage

Equitable access to medicines and healthcare is enshrined in the World Health Organization’s UHC concept, which is defined as follows^6^:

Universal health coverage means that all people have access to the health services they need, when and where they need them, without financial hardship. It includes the full range of essential health services, from health promotion to prevention, treatment, rehabilitation, and palliative care. (n.p.)

The concept of health systems strengthening comprises the policy tools required to attain this goal of UHC.^7^ Currently, South Africa is an upper-middle-income country^8^ moving towards UHC through the National Health Insurance (NHI) policy funding initiative. The NHI is meant to bridge the gap in inequity in healthcare currently still experienced in South Africa because of the existence of a two-tiered public and private healthcare system. The NHI bill^9^ was passed on 08 August 2019. For South Africa the policy dialogue now needs to shift from critiquing the contents of the NHI policy to a conversation around the reasons why there is still inequity in medicines access in South Africa. The shortfalls of the existing healthcare and essential medicines policies need to be investigated to make recommendations towards achieving access to medicines under UHC. One of the National Drug Policy tools meant to address access to medicines inequity is the STGs/EML, which still features prominently in the new NHI package of benefits.^9^ However, since its implementation in 1996, an evaluation of the impacts of the STGs/EML on availability, affordability and pricing of medicines in South Africa has not been done. Studies conducted thus far that quantitatively analysed changes in the South African STGs/EML (2016) and interviewed members from the NEMLC on the selection of medicines (2017) found that the monitoring and evaluation of the South African STGs/EML policies and processes were wanting over the years.^10,11^ This means that the successes and/or failures of the essential medicines policies and STGs/EML and their impacts on the end users or patients in the public sector have possibly not been highlighted. Strengthening health systems comprises ‘a significant, purposeful effort to improve performance’^12^ and goes further than investing inputs as it also involves reforming how the healthcare system currently operates.^13^ Despite the efforts of the new democratic government to ensure equitable access to medicines to all by introducing the STGs/EML into the public healthcare system, many questions surrounding the South African STGs/EML processes and impact thereof remain unanswered and warrant investigation. This is especially important in light of the fact that the STGs/EML is to be effectively utilised in the NHI financing system.

## Lessons for national health insurance in South Africa

The following knowledge gaps exist and must be filled to strengthen South Africa’s healthcare system. These suggestions are designed to improve transparency in the policies and processes currently in operation and to further provide recommendations for possible improvement where required.

An investigation into how the healthcare budget is calculated for the burden of disease and how this is translated into pharmaceutical expenditure for STGs/EML as well as for non-STGs/EML items must be done. There is currently dispensation to national and then provincial departments of health who have autonomy in terms of their budget allocation and expenditure for medicines, but this is not explicit nor is this public knowledge. Understanding the allocation process towards medicines expenditure will assist the government and other stakeholders to develop cost-benefit packages for NHI. Another model would be to ring-fence the medicines budget from benefit packages that consider all other interventions and care pathways.

The essential drugs programme is a branch of the affordable medicines directorate responsible for the selection and review of essential medicines (done by the NEMLC) and the rational use of medicines.^14^ The financial implications of having STGs/EMLs in South Africa must also be investigated to make transparent the actual costs of reviewing and updating STGs/EMLs and how this process is funded. This needs to be researched, as it is not known if the processes and policies used in the daily functioning of the essential drugs programme are cost-efficient and/or sustainable. What are the capacity-building and succession-planning initiatives and policies of the NEMLC surrounding the running of the essential drugs programme? Should training be instituted across health sciences programmes in academic institutions at undergraduate or postgraduate level in order to build a community of practitioners around evidence-based guideline development? Moreover, are the desired effects of the essential drugs programme policies being achieved? Is the NEMLC monitoring and evaluating its functioning against their operational plans and policies and timeously implementing effective corrective measures? Widespread media reports of medicine shortages at public health facilities and the rising costs of medicines make monitoring of the essential drugs programme critical. In an NHI environment, this would need to be considered in terms of whether a national committee structure would be more efficient and cost-effective in terms of reviewing guidelines for treatment under benefit packages. Lessons from the current NEMLC approach can be used to guide NHI structures and their development. With an increasing emphasis on health technology assessment (HTA) use in UHC roll-out, the application of HTA principles by the NEMLC and its related task teams will need to be identified. The practical application of HTA in a South African environment, with information and data constraints, will be useful in NHI. Plans will need to be developed, more data gathered, registries constructed and transparency extended about assumptions made in modelling under HTA. Principles used to balance the outcomes of HTA with budget impact analyses can also be formulated based on the NEMLC experiences.

The gaps that have been identified therefore require further investigation, and recommendations should be made to improve the efficiency of the country’s essential drugs programme. An updated trend analysis of changes to the South African STGs/EML and evaluation of these impacts may aide this process. Furthermore, to provide a global picture, it would be interesting to investigate how South Africa’s EML compares with other countries (in Africa and around the world). Comparisons could also be made regarding the progress with UHC in terms of policies and processes for medicine listings, review and updating, and monitoring and evaluation of EMLs. These country case studies could be possible exemplars for South Africa, or to the contrary, the South African situation could provide lessons for other countries attempting to use the essential medicines concept and expanding this under UHC policies.

## Conclusion

With the NHI bill just being passed, much research is required to fill these gaps and provide new knowledge in the South African context and to provide recommendations to inform not only pharmaceutical and essential medicines policy development but also for the NHI policy implementation. Currently, this type of policy evaluation is scarce in South Africa, and without a greater investment in strengthening the health system from a policy perspective, it may be difficult to achieve the goals of the NHI and ultimately those for UHC.
